# Supplementary figures and images for: The chromatin landscape of primary synovial sarcoma organoids is linked to specific epigenetic mechanisms and dependencies
Source: Life Sci Alliance. 2020 Dec 23;4(2):e202000808. doi: 10.26508/lsa.202000808 (PMC7768195; doi:10.26508/lsa.202000808)

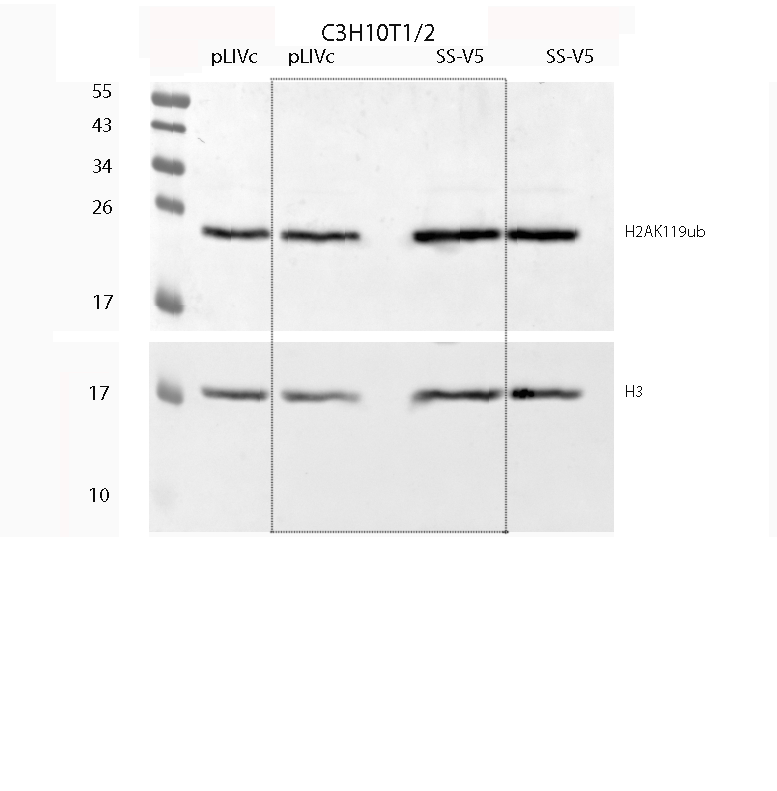

Supplement: Supplementary file 1 [file LSA-2020-00808_SdataFS7.1.tif]

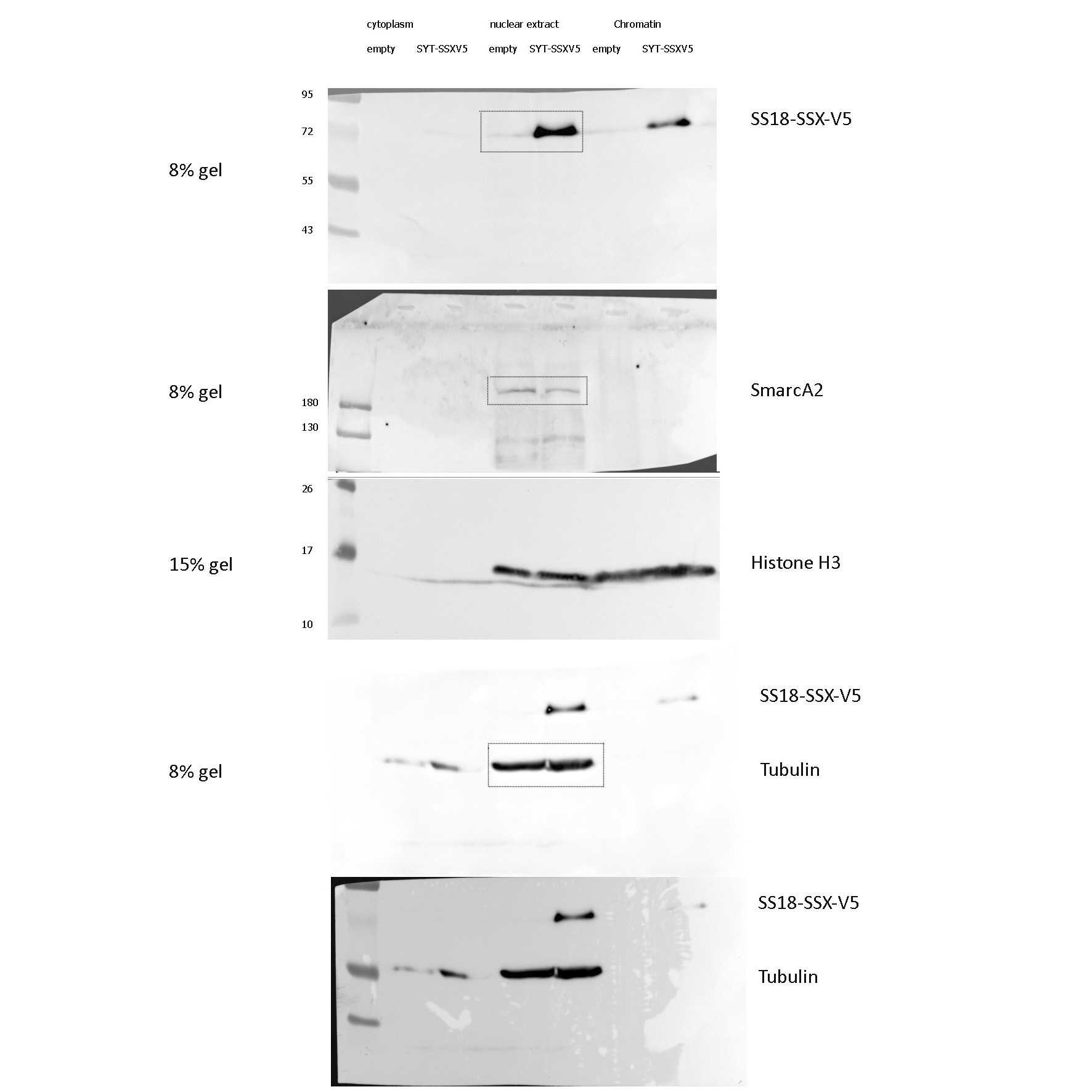

Supplement: Supplementary file 2 [file LSA-2020-00808_SdataFS7.2.tif]
